# Morphological and genetic evidence for the *Sarcaulus brasiliensis* complex (Sapotaceae, Chyrsophylloideae) reveals a new species from the rainforests of the Middle Magdalena Valley, Colombia

**DOI:** 10.3897/phytokeys.273.175192

**Published:** 2026-04-09

**Authors:** M. Alejandra Jaramillo, Terence D. Pennington, Gerardo A. Aymard-Corredor, Andrés F. Majin-Ladino

**Affiliations:** 1 Grupo Diversitas, Facultad de Ciencias Básicas y Aplicadas, Universidad Militar Nueva Granada, km 2 vía Cajicá-Zipaquirá, Cundinamarca, Colombia Field Museum of Natural History Chicago United States of America; 2 Negaunee Integrative Research Center, Field Museum of Natural History, Chicago, USA Universidad Militar Nueva Granada Cajicá-Zipaquirá Colombia; 3 Royal Botanic Gardens, Kew, Richmond, Surrey, UK Royal Botanic Gardens Kew United Kingdom; 4 UNELLEZ-Guanare, Programa de Ciencias del Agro y el Mar, Herbario Universitario (PORT), Mesa de Cavacas, estado Portuguesa 3350, Venezuela Herbario Universitario (PORT) Mesa de Cavacas Venezuela

**Keywords:** Chocó Region, Ericales, Flora Neotropical, Flora of Colombia, Ericales, Flora Neotropical, Flora de Colombia, Región del Chocó, *
Sarcaulus
*

## Abstract

*Sarcaulus* is a small genus of neotropical trees in the Sapotaceae. Most specimens in herbaria are identified as *S. brasiliensis*, a species recognized from Costa Rica to Bolivia. Morphological characteristics and molecular sequence data help us to identify a new species of *Sarcaulus* with hermaphrodite flowers from the Middle Magdalena Valley, Colombia. A complete description, an illustration, a Lancaster plate, information about habitat, phenology, conservation status, and the species name etymology are presented. In addition, a key for identifying *Sarcaulus* species is provided. *Sarcaulus paujilensis* is remarkable for its bisexual flowers, in an otherwise predominantly unisexual (plant dioecious) genus. This new finding increases the number of species of the genus in the Neotropical flora to six. More fieldwork and molecular phylogenetics are needed to establish many new taxa.

## Abstract (es)

*Sarcaulus* es un pequeño género neotropical de árboles de la familia Sapotaceae. La mayoría de los especímenes en los herbarios se han identificado como *Sarcaulus brasiliensis*, una especie reconocida desde Costa Rica hasta Bolivia. El estudio de las características morfológicas y de las secuencias moleculares ayudó a identificar una nueva especie de *Sarcaulus*, con flores bisexuales del Valle Medio del Magdalena, en Colombia. Se presenta una descripción completa, una ilustración, una lámina de Lancaster, información sobre el hábitat, la fenología, el estado actual de conservación, la etimología del nombre de la especie y una clave para identificar las especies de *Sarcaulus*. *S. paujilensis* destaca por sus flores bisexuales, en un género predominantemente con flores unisexuales (plantas dioicas). Este nuevo hallazgo aumenta el número de especies del género a seis. Más trabajo de campo, en los herbarios y en la filogenética molecular, permitirá establecer nuevos taxones de este género.

## Introduction

Sapotaceae is a mid-size pantropical family that comprises trees and shrubs that are widely distributed throughout the tropics, subtropics, and far places such as the Austro mediterranean biomes at the coastal regions of Chile [i.e., *Gayella valparadisaea* (Molina) Pierre; *sensu*: Pennington (1990); Swenson et al. (2023a)], ca. 1300 species in 74 genera (Swenson et al. 2023b). Of them, 21 genera and ca. 500 species are present in the Neotropics (Swenson et al. 2020, 2023a). A large number of taxa occur in lowland and low-montane rainforests, with some species extending to the coastal plains, dunes, mangroves, and seasonally tropical dry forests (*sensu*: Pennington 1990). Many Sapotaceae species are economically important (Petersen et al. 2012) and represent an essential component of the forest flora, both in terms of species diversity and individual abundance (i.e., *Micropholis guyanensis* (A. DC.) Pierre, *M. gardneriana* (A. DC.) Pierre, *Ecclinusa guianensis* s.l. Eyma) in lowland neotropical forests of the Amazon basin (Pennington 1990, 2004; ter Steege et al. 2023).

Molecular phylogenetics studies in Sapotaceae support three subfamilies: Chrysophylloideae, Sapotoideae, and Sarcospermatoideae (Swenson and Anderberg 2005). The generic limits within the subfamilies are not settled, ranging from 122 genera recognized by Aubréville (1962) to 53 by Pennington (1990). Phylogenetic analyses have shown that the large genera *Chrysophyllum* and *Pouteria* are polyphyletic as circumscribed earlier (Baehni 1942; Aubréville 1962; Pennington 1990), partly supporting Aubréville’s classification with many small genera. According to the latter author, there are no clear-cut diagnostic characters between *Pouteria* and *Chrysophyllum*, and he proposed recognizing several separate genera already described earlier by Pierre, Baillon, and others (Aubréville 1962). Swenson et al. (2023b) proposed reinstating various genera and redefining the limits of *Chrysophyllum* and *Pouteria* s.s. Even though: a) many *Pouteria* species are provisionally left as *Pouteria* s.l., and b) there is a recent proposal to synonymize the genus *Sarcaulus* under *Pouteria* s.l. (De Faria et al. 2017). However, *Swenson* et al. (2023b) maintained *Sarcaulus* as a distinct genus (despite its monophyly not having been tested).

*Sarcaulus* Radlk. is a small genus of Sapotaceae, occurring in neotropical lowland and lower montane rain forests distributed from Costa Rica to Bolivia, reaching Surinam (absent in Venezuela) and the Atlantic Forest of Brazil (Pennington 1990, 2004). The genus is easily distinguished from other Sapotaceae genera by its distichous leaves, swollen corolla, and unisexual flowers (dioecious trees). The flowers are rarely seen, which makes species identification difficult. Most specimens of *Sarcaulus* are identified as *S. brasiliensis* (A.DC.) Eyma, a species distributed throughout Costa Rica and South America. While the other species in the genus are confined to the Amazon basin, *S. inflexus* (A.C. Sm.) T.D. Penn. and *S. vestitus* (Baehni) T. D. Penn. is distributed in the western Amazon of Brazil, Ecuador, and Peru, *S. oblatus* T.D. Penn. is only known from eastern Ecuador. *S. wurdackii* Aubr. is restricted to the Peruvian Amazon (known only from the type collection).

The genus *Sarcaulus* was included in the treatment of Sapotaceae for Flora Neotropica (Pennington 1990), Flora Mesoamericana (Pennington et al. 2009), “Manual de plantas de Costa Rica” (Morales 2015), and in the “Catálogo de plantas y líquenes de Colombia” (Pennington and Bernal 2016). However, currently, a considerable number of specimens exist that have been identified only to genus in many large herbaria (F, K, MO, NY, US; acronyms follow Thiers 2025, continuously updated). Perhaps this is due to the significant morphological variation and the wide distribution encompassed by *S. brasiliensis* that suggest there is a need to study the genus in detail because some new species might be hidden within this “variable” taxon. The last species of *Sarcaulus* described dates back 36 years to the Flora Neotropica (Pennington 1990).

Lowland tropical rainforests harbor the world’s most diverse flora (Draper et al. 2019, 2021; Cazzolla Gatti et al. 2022). In South America, this biome is especially rich in species in the Amazon and the Chocó Region. Despite its richness, our knowledge of its tree species and genera remains relatively limited (ter Steege et al. 2020, 2023; Luize et al. 2024). Compared to the Amazon, the lowland rainforests on the other side of the Andes cover a smaller area, and we know much less about their ecology, flora, and vegetation types. Therefore, improving the delimitation of genera such as *Sarcaulus* and their species will significantly contribute to our understanding of the diversity of neotropical trees.

The Chocó Region extends from wet Central America to northern Ecuador, west of the Andes, and eastward to the Middle Magdalena Valley (Bocanegra-González et al. 2024). The Middle Magdalena Valley in Colombia comprises lowland and lower montane rainforest ecosystems. The area’s rich floral diversity has largely gone unexplored due to armed conflicts. Still, renewed interest in the region has led to the description of many new species (Aymard Corredor and Jaramillo 2023; Mendoza-Cifuentes et al. 2023; Aymard Corredor et al. 2024; Majín-Ladino et al. 2024; Londoño-Echeverri 2025). We describe and illustrate a new species of *Sarcaulus* from the lowland forests of the Middle Magdalena Valley and propose a new key to identify the taxa of the genus. The new species was identified during fieldwork conducted by the Herbarium of Universidad Militar Nueva Granada, which has been actively studying the region’s flora in recent years.

## Materials and methods

We examined in person herbarium specimens from Colombia deposited in the collections COAH, COL, and JAUM. In addition, all type specimens, as well as general collections hosted by virtual herbaria, were consulted, including those maintained by the Field Museum (F; http://emuweb.fieldmuseum.org/botany/taxonomic.php), JSTOR Global Plants (http://plants.jstor.org), Missouri Botanical Garden (https://www.tropicos.org), and Smithsonian Institution (US; https://collections.si.edu/search/).

This work is based on morphological and herbarium studies. The species description was based on field observations (with flower and fruit material preserved in ethanol) and herbarium specimens. The flowers from herbarium specimens were rehydrated for three days before measurement in a 1:1 glycerin-0.9% NaCl solution. Terminology for vegetative characters, inflorescences, flowers, and fruit morphology follows Pennington (1990, 2009) and Font-Quer (2001).

We extracted DNA from herbarium specimens using the QIAGEN DNeasy Plant Mini Kit (Qiagen, Valencia, California) and the CTAB method. The ITS region was amplified using standard primers (Baldwin 1992). Sequencing was contracted with Gencell Pharma (Bogotá, Colombia). Leaf tissue from other *Sarcaulus* species was unavailable or of poor quality; thus, we were unable to include other species of the same genus in the phylogenetic analysis. The resulting sequences were aligned using MAFFT (Katoh and Standley 2013). We selected 75 ITS sequences from GenBank to evaluate the relationships of the new species of Sapotaceae. We used *Donella lanceolata* (Blume) Aubrév. from Madagascar as the outgroup, following previous analyses of Chrysophyllidae (Swenson et al. 2023b). A total of 79 Sapotaceae sequences, comprising representatives of 11 genera (sensu Swenson et al. 2023b), were used to identify the relationships of the new taxon. Maximum Likelihood (ML) phylogenetic analyses were conducted using RAXML (Stamatakis 2014). Clade support was tested using 1000 bootstrap replicates as implemented in RAXML.

## Results and discussion

### Phylogenetic relationships

**Figure 1.**
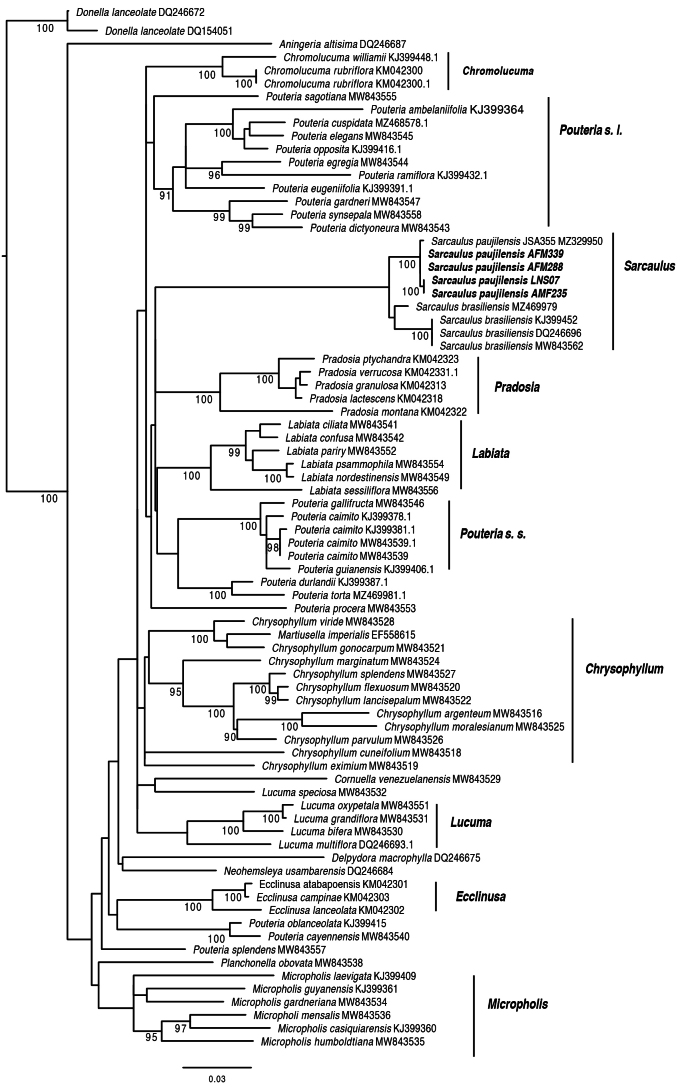
Phylogenetic relationships of *Sarcaulus paujilensis* (in bold, new sequences produced for this study). Numbers above branches are Bootstrap values > 90.

Four new sequences of ITS for the new taxon were obtained and aligned to existing sequences of Sapotaceae (Table 1). A total of 79 sequences and 905 nucleotide sites were aligned for the analysis. The phylogenetic relationships retrieved are consistent with previous global analyses of the family (Swenson et al. 2023b). Sequences from the new taxon are nearly identical with an estimated intraspecific variation of ca. 0.3%, in contrast to the interspecific variation of 2.28–3.5% genetic distance. These molecular sequence differences agree with morphological and geographic differentiation. The sequences we produced form a strongly supported clade (BS = 100), including a previously published sequence identified as “*Sarcaulus brasiliensis*” but collected in the middle Magdalena Valley (Serrano et al. 2021). The above monophyletic clade is sister to a clade including *S. brasiliensis* sequences from the Amazon region (Fig. 1). The phylogenetic relationships and morphological characteristics confirm that we have found a new species of *Sarcaulus*. Here, we follow the nomenclatural proposal by Swenson et al. (2023b). Notably, our study provides additional evidence for the monophyly of the genus.

**Table 1.** GenBank accessions for new species and reference taxa. Collections according to the Index Herbariorum (acronyms follow Thiers 2025, continuously updated). New species in bold.

Species	GenBank accession ITS	Voucher	Collection	Publication
*Aningeria altissima* (A. Chev.) Aubrév. & Pellegr.	DQ246687.1	I. Friis, M.G. Gilbert & K. Vollesen 4145	UPS	Swenson et al. 2008
*Chromolucuma rubriflora* Ducke	KM042300	Terra-Araujo 833	INPA, S	Terra-Araujo et al. 2015
*Chromolucuma williamii* (Aubrév. & Pellegr.) Alves-Araújo	KJ399448.1	Brazil Souza & al. 477	INPA	Terra-Araujo et al. 2015
*Chrysophyllum argenteum* Jacq.	MW843516	Miller 9238	INPA, S	Swenson et al. 2023b
*Chrysophyllum cuneifolium* A.DC.	MW843518	Poncy 1730	P, S	Swenson et al. 2023b
*Chrysophyllum eximium* Ducke	MW843519	Poncy 1835	P, S	Swenson et al. 2023b
*Chrysophyllum flexuosum* Mart.	MW843520	Stefani 47	S, UEC	Swenson et al. 2023b
*Chrysophyllum gonocarpum* (Mart. & Eichler ex Miq.) Engl.	MW843521	Zardini 44475	MO, S	Swenson et al. 2023b
*Chrysophyllum lancisepalum* R. Lima	MW843522	Popovkin 1499	P	Swenson et al. 2023b
*Chrysophyllum marginatum* (Hook. & Arn.) Radlk.	MW843524	Pavão s. n.	FUEL, S	Swenson et al. 2023b
*Chrysophyllum moralesianum* Aguilar, D.Santam. & J.M.Chaves	MW843525	Anderberg 31	S	Swenson et al. 2023b
*Chrysophyllum parvulum* Pittier	MW843526	Madsen 7823	AAU, LOJA	Swenson et al. 2023b
*Chrysophyllum splendens* Spreng.	MW843527	AA 733	S, UFP	Swenson et al. 2023b
*Chrysophyllum viride* Mart. & Eichler ex Miq.	MW843528	Verdi 670	FURB, S	Swenson et al. 2023b
*Cornuella venezuelanensis* Pierre	MW843529	Faria 2008	FUEL, S	Swenson et al. 2023b
*Delpydora macrophylla* Pierre	DQ246675	Wieringa & Haegenes 2092	WAG	Swenson et al. 2008
*Donella lanceolata* (Blume) Aubrév.	DQ246672	I.V. Bartish & A. Ford 34	BRI, S	Swenson et al. 2008
*Donella lanceolata* (Blume) Aubrév.	DQ154051	I.V. Bartish & A. Ford 34	BRI, S	Swenson et al. 2008
*Ecclinusa atabapoensis* (Aubrév.) T.D.Penn.	KM042301	Costa 486	INPA	De Faria et al. 2017
*Ecclinusa campinae* Terra-Araujo & F.M.Costa	KM042303	Costa 1209	INPA	De Faria et al. 2017
*Ecclinusa lanceolata* Pierre	KM042302	Terra-Araujo 542	INPA	De Faria et al. 2017
*Labiata ciliata* (Alves-Araújo & M.Alves) Alves-Araújo	MW843541	Silva 67	not available	Swenson et al. 2023b
*Labiata confusa* (Alves-Araújo & M.Alves) Alves-Araújo	MW843542	Pirani 2715	NY	Swenson et al. 2023b
*Labiata nordestinensis* (Alves-Araújo & M.Alves) Alves-Araújo	MW843549	AA 1259	CEPEC, UFP, VIES	Swenson et al. 2023b
*Labiata pariry* (Ducke) Alves-Araújo	MW843552	Krukoff 5034	S	Swenson et al. 2023b
*Labiata psammophila* Mart.	MW843554	Cardoso 1681	RB, S	Swenson et al. 2023b
*Labiata sessiliflora* Sw.	MW843556	Zanoni 47094	S	Swenson et al. 2023b
*Lucuma bifera* Molina	MW843530	Faria 2014/22	FUEL, S	Swenson et al. 2023b
*Lucuma grandiflora* A.DC.	MW843531	Jost 398	ALCB	Swenson et al. 2023b
*Lucuma multiflora* A.DC.	DQ246693.1	Villa 257	BM	Swenson et al. 2008
*Lucuma oxypetala* (T.D.Penn.) Swenson	MW843551	Fiaschi 2506	CEPEC, NY	Swenson et al. 2023b
*Lucuma speciosa* Ducke	MW843532	Ribeiro 2899	FUEL, S	Swenson et al. 2023b
*Martiusella imperialis* (Linden ex K.Koch & Fintelm.) Pierre	EF558615	Pennington s. n.	S	Bartish et al. 2011
*Micropholis mensalis* (Baehni) Aubrév.	MW843536	Munzinger 1804	CAY, P, S	Swenson et al. 2023b
*Micropholis casiquiarensis* Aubrev.	KJ399360	Brazil Nascimento & al. 770	INPA	De Faria et al. 2017
*Micropholis gardneriana* Pierre	MW843534	AA 1236	UFP	Swenson et al. 2023b
*Micropholis guyanensis* Pierre	KJ399361	Brazil Hopkins & al. 1475	INPA	De Faria et al. 2017
*Micropholis humboldtiana* (Roem. & Schult.) T.D.Penn.	MW843535	Damasco 1224	INPA, S	Swenson et al. 2023b
*Micropholis laevigata* (Mart.) Swenson & A.D.Faria	KJ399409	Brazil Vicentini & al. 762	INPA	De Faria et al. 2017
*Neohemsleya usambarensis* T.D.Penn.	DQ246684	Borhidi & al. 84905	UPS	Swenson et al. 2008
*Planchonella obovata* (R.Br.) Pierre	MW843538	Swenson 2111	NY, S, VNMN	Swenson et al. 2023b
*Pouteria ambelaniifolia* (Sandwith) T.D.Penn.	KJ399364	Brazil Ribeiro & al. 1895	INPA	De Faria et al. 2017
*Pouteria caimito* (Ruiz & Pav.) Radlk.	KJ399378.1	Brazil Manaus Assuncao & Silva 649	INPA	De Faria et al. 2017
*Pouteria caimito* (Ruiz & Pav.) Radlk.	KJ399381.1	Brazil Ubatuba Bertoni & Geremias 295	IAC	De Faria et al. 2017
*Pouteria caimito* (Ruiz & Pav.) Radlk.	MW843539.1	AA 1203	CEPEC, UFP	Swenson et al. 2023b
*Pouteria caimito* (Ruiz & Pav.) Radlk.	MW843539	AA 1203	CEPEC, UFP	Swenson et al. 2023b
*Pouteria cayennensis* Eyma	MW843540	Mori 23200	CAY, K	Swenson et al. 2023b
*Pouteria cuspidata* (A.DC.) Baehni	MZ468578.1	J. Serrano 176	not available	Serrano et al. 2021
*Pouteria dictyoneura* Radlk.	MW843543	E.L. Ekman 6100	S	Swenson et al. 2023b
*Pouteria durlandii* (Standl.) Baehni	KJ399387.1	Brazil Ribeiro & al. 1904	INPA	De Faria et al. 2017
*Pouteria egregia* Sandwith	MW843544	J.J. Wurdack & J.V. Monachino 39693	S	Swenson et al. 2023b
*Pouteria elegans* (A.DC.) Baehni,	MW843545	F.M. Costa & al. 955	INPA, S	Swenson et al. 2023b
*Pouteria eugeniifolia* (Pierre) Baehni	KJ399391.1	Brazil Faria and Ribeiro 2007/38	INPA	De Faria et al. 2017
*Pouteria gallifructa* Cronquist	MW843546	AA 1245	UFP	Swenson et al. 2023b
*Pouteria gardneri* (Mart. & Miq.) Baehni	MW843547	A. Alves-Araújo & S. Martins 1116	S, UFP	Swenson et al. 2023b
*Pouteria guianensis* Aubl.	KJ399406.1	Brazil Ribeiro & al. 1902	INPA	De Faria et al. 2017
*Pouteria oblanceolata* Pires	KJ399415	Brazil Ribeiro & Silva 1373	INPA	De Faria et al. 2017
*Pouteria opposita* (Ducke) T.D.Penn.	KJ399416.1	Brazil Martins & al. 45	INPA	De Faria et al. 2017
*Pouteria procera* (Mart.) T.D.Penn.	MW843553	Faria 2014	FUEL, S	Swenson et al. 2023b
*Pouteria ramiflora* Radlk.	KJ399432.1	Brazil Faria & Ribeiro 2008/5	SPF	De Faria et al. 2017
*Pouteria sagotiana* (Baill.) Eyma	MW843555	Jansen 5475	NY, U	Swenson et al. 2023b
*Pouteria splendens* (A.DC.) Kuntze	MW843557	Luebert 3128	BONN, S	Swenson et al. 2023b
*Pouteria synsepala* Popovkin & A.D.Faria	MW843558	A.V. Popovkin & C.J. Mendes 1755	FUEL, HUEFS, S	Swenson et al. 2023b
*Pouteria torta* Radlk.	MZ469981.1	Anderberg et al. 47	not available	Serrano et al. 2021
*Pradosia granulosa* Pires & T.D.Penn.	KM042313	Terra-Araujo 86	INPA, S	Terra-Araujo et al. 2015
*Pradosia lactescens* Radlk.	KM042318	Terra-Araujo 764	INPA, S	Terra-Araujo et al. 2015
*Pradosia montana* T.D.Penn.	KM042322	Palacios & Rubio 9968	NY	Terra-Araujo et al. 2015
*Pradosia ptychandra* (Eyma) T.D.Penn.	KM042323	Mori 25441	S	Terra-Araujo et al. 2015
*Pradosia verrucosa* Ducke	KM042331.1	Terra-Araujo 780	INPA, S	Terra-Araujo et al. 2015
***Sarcaulus paujilensis* this study**	MZ329950	J. Serrano 355	not available	Serrano et al. 2021
***Sarcaulus paujilensis* this study**	**PV599987.1**	**A. Majin-Ladino 339**	**UMNGH**	**this study**
***Sarcaulus paujilensis* this study**	**PV599990.1**	**A. Majin-Ladino 288**	**UMNGH**	**this study**
***Sarcaulus paujilensis* this study**	**PV599989.1**	**A. Majin-Ladino 235**	**UMNGH**	**this study**
***Sarcaulus paujilensis* this study**	**PV599988.1**	**L. Suarez 07**	**UMNGH**	**this study**
*Sarcaulus brasiliensis* Eyma	MZ469979	Richardson et al. 322	not available	Serrano et al. 2021
*Sarcaulus brasiliensis* Eyma	KJ399452	Brazil Martins et. al. 48	INPA	De Faria et al. 2017
*Sarcaulus brasiliensis* Eyma	DQ246696	Paniagua et al. 4852	MO	Swenson et al. 2008
*Sarcaulus brasiliensis* Eyma	MW843562.1	Paniagua 4852	MO	Swenson et al. 2023b

### Taxonomic treatment

**Figure 2.**
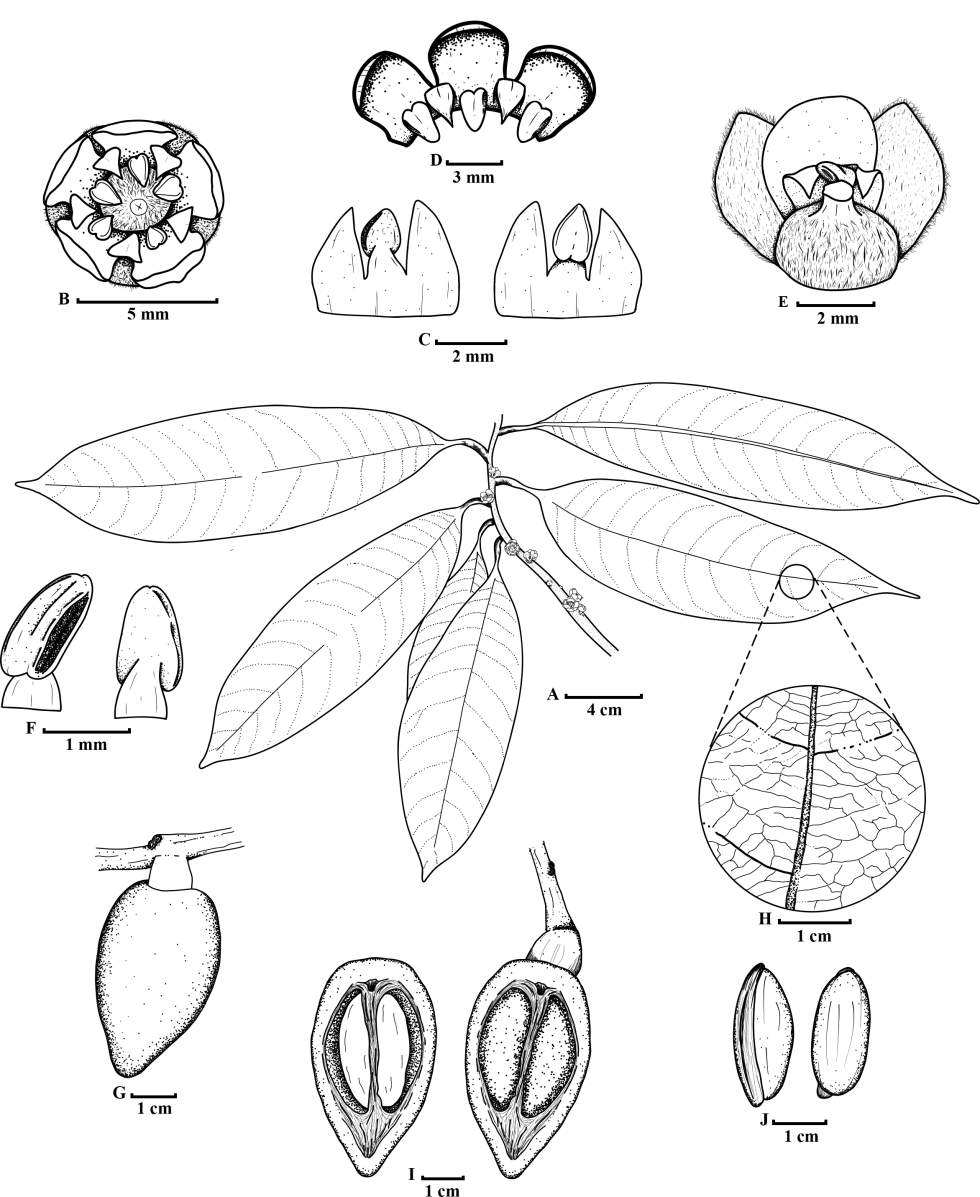
*Sarcaulus paujilensis*. **A**. Flowering branch; **B**. Flower from the above; **C**. stamens and staminodes; **D**. Petals, stamens and staminodes; **E**. Stamens; **F**. Pistil and perianth; **G**. Fruit; **H**. Detail of leaf tertiary venation; **I**. Fruit interior; **J**. seeds—illustration by Andrés F. Majín-Ladino.

**Figure 3.**
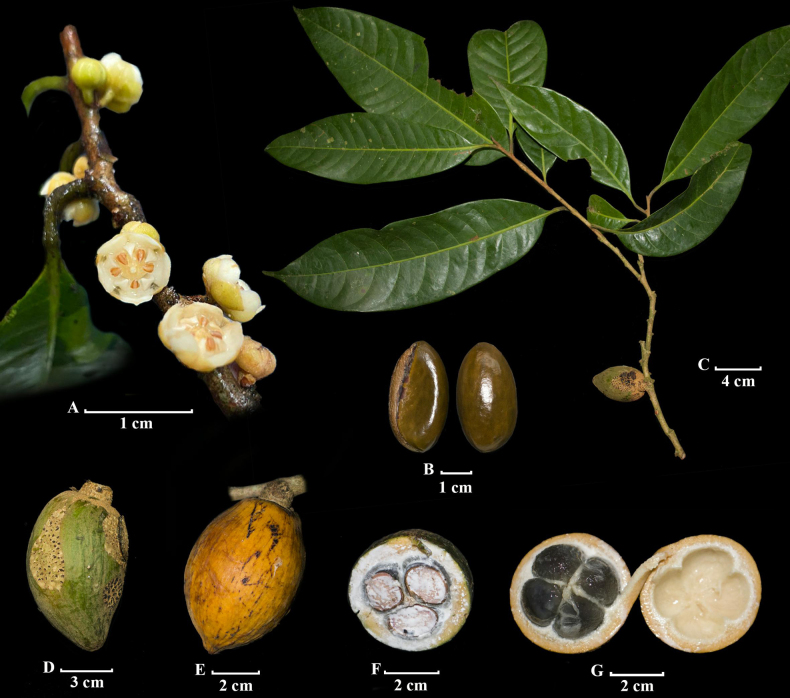
*Sarcaulus paujilensis*. **A**. Flowering branch; **B**. Seed; **C**. Fruiting branch; **D**. Immature fruit; **E**. Mature fruit; **F, G**. Transversal section of fresh fruit. Photos by **A**. Lizette Sierra, Paula Lara, and Luisa Suarez; **B–G**. Andrés F. Majín-Ladino.

We provide an emended description of the genus, noting that the flowers of this new species are bisexual (Figs 2, 3).

#### Sarcaulus

Taxon classificationPlantaeEricalesSapotaceae

Radlkofer, Sitzungsber. Math.- Phys. Cl.Königl. Bayer. Akad. Wiss. Miinchen 12: 310. 1882.

CECEA564-5293-5597-93FC-C7983D03C328

##### Type.

*Sarcaulus macrophyllus* (Martius) Radlkofer (=*Chrysophyllum macro*-*phyllum* Martius).

##### Description.

***Trees*** to 30 m high. ***Leaves*** spaced, slightly spirally arranged or alternate and distichous, venation eucamptodromous or brochidodromous. ***Flowers*** unisexual (plant dioecious) or bisexual. ***Calyx*** has a single whorl of five sepals. ***Corolla*** globose, broadly cyathiform or subrotate, tube and/or petals weakly to strongly carnose, corolla tube equaling or slightly exceeding the lobes; lobes usually five, slightly imbricate or subvalvate. ***Stamens*** five, fixed at the top of the corolla tube, exserted; filaments short, swollen; anthers strongly inflexed. ***Staminodes*** five, thick, carnose, erect, or incurved against the style. Disk absent. ***Ovary*** 1–5-locular. ***Fruit*** a berry. ***Seeds*** 1-several, laterally compressed, testa smooth or wrinkled, shining; scar adaxial, full-length; embryo with plano-convex, free cotyledons, radicle extending to the surface; endosperm absent.

#### Sarcaulus paujilensis

Taxon classificationPlantaeEricalesSapotaceae

M.A.Jaram. & T. D.Penn. sp. nov.

BD19A09B-3873-572E-9DD0-339DDC490EE6

urn:lsid:ipni.org:names:77378402-1

##### Type.

Colombia • Boyacá, Municipio Puerto Pinzón, Reserva Proaves El Paujil, 6°03'01"N, 74°15'51"W, 200 m, 9 May. 2024, [fl], *Lizette Sierra, Paula Lara & Luisa Suarez LNS00-7* (Holotype: UMNG-H). Figs 2, 3.

##### Diagnosis.

*Sarcaulus paujilensis* resembles *S. oblatus*, however, this new species can be differentiated from the latter by having leaves oblanceolate to oblong (vs. elliptic to narrowly oblong-elliptic), petiole 1.7–2.5 cm long (vs. 8–15 mm long), flowers paired (vs. fascicles 5-many-flowered), bisexual (vs unisexual), sepals 5–6 mm (vs. 2.5–3 mm), staminodes ca. 2 mm long (vs. 0.5–1 mm), ovary 1–4-locular, shortly sericeous (vs. 4­5-locular, minutely adpressed puberulent), fruits ca. 50 × 25–30 mm, ovoid (vs. 22–25 × 30–32 mm, oblate), and seeds 25–28 mm long (vs. seeds 17–18 mm long).

##### Description.

***Tree*** to 15–40 m tall; young branches pilose, becoming glabrous when mature. ***Leaves*** spaced, slightly spirally arranged, appearing distichous, 21–26 × 6.0–7.1 cm, oblanceolate to oblong, apex shortly acuminate, base acute, coriaceous, glabrous on both surfaces; venation brochidodromous, midrib and secondary veins sunken on the adaxial surface, secondary veins 12–14 pairs, parallel, arcuate; intersecondaries absent; tertiaries reticulate. ***Petioles*** 1.7–2.5 cm long, channeled, glabrous. ***Inflorescences*** of paired flowers, axillary. ***Pedicel*** ca. 3 mm long, densely ochreous sericeous. ***Flowers*** bisexual. ***Sepals*** five, 5–6 × 4–5 mm, broadly ovate, apex acute–obtuse, appressed ochreous sericeous on both surfaces. ***Corolla*** cyathiform, lobes carnose, fused 1.0–1.3 mm, lobes 4–6 × 3–4 mm, ovate, apex obtuse, glabrous on both surfaces, margin papillate. ***Stamens*** 5; fixed at ca. 1.0 mm from the base of the corolla lobes, filaments ca. 1.0 mm long, not geniculate, glabrous; anthers 1.75–2.0× ca. 1 mm, cordate-deltate, glabrous. ***Staminodes*** 5, ca. 2 mm long, triangular. Disk absent. ***Ovary*** globose with a truncate apex, 1–4 locules, shortly sericeous; style 0.8–0.9 mm long during anthesis, glabrous; style-head capitate. ***Fruits*** ca. 5 × 2.5–3 cm, green immature, yellow-orange to mature, ovoid, apex acute, smooth, sericeous ochre, thickened receptacle at the base of the fruit, 7–8× ca. 10 mm. ***Seeds*** 1 to 4, 25–28 × 10–12 mm, shape oblong-elliptic, laterally compressed, testa smooth and glossy; scar 4–5 mm wide, oblong, extending the length of the seed; endosperm absent.

##### Phenology.

Specimens in flower were collected in May, in fruit in October, and in February.

##### Eponomy.

*Sarcaulus paujilensis* is named after Reserva Proaves “El Paujil”. We honor the efforts of the reserve personnel, who have continuously protected the biodiversity of the Middle Magdalena Valley lowland and lower-montane rainforest for over 20 years.

##### Distribution and habitat.

The species is known to occur in lowland rainforest remnants at elevations of 200–300 m.

##### Preliminary conservation status.

This species is currently known only from the type locality and three specimens from the same area. However, under IUCN (2024) guidelines, there is insufficient data to accurately determine its conservation status; it is listed as Data Deficient (DD) and reported here as a rare species. Although conservation status assessments can be made for species with such small numbers of collections (Rivers et al. 2011), it may be difficult to determine whether the appearance of rarity in a species is due to the lack of, or outdated, data, collection artifact, or to its actual rarity (Verspagen and Erkens 2022).

The conservation of these forests is at risk due to ongoing deforestation in the middle Magdalena Valley, especially from 1985 to 2000 (Rodríguez Eraso et al. 2013) and in recent years (Salgado et al. 2022). Deforestation, degradation, and water pollution continue to expand (Salgado et al. 2022), driven by significantly greater agricultural use, pasture, selective logging, illicit crops, and mining. Fortunately, the area where *Sarcaulus paujilensis* occurs is part of the Reserva Proaves El Paujil, a private reserve designed to protect bird diversity and safeguard the forests where the birds occur.

##### Taxonomic notes.

This new species is morphologically most similar to *Sarcaulus oblatus* (Pennington, 1990). Both species have glabrous leaves and relatively short pedicels, ca. 1.5–4 mm long, compared to more than 10 mm long in the other species. *S. oblatus* is endemic to the NW Amazon of Ecuador, while *S. paujilensis* is found across the Andes in the middle Magdalena Valley. *S. paujilensis*, has been previously misidentified as *S. brasiliensis* (Serrano et al. 2021). This highlights the importance of fieldwork in the genus. *Sarcaulus* is easily recognizable by the leaf disposition; however, as the taxon flowers rarely, many samples are assigned to the morphologically diverse and broadly distributed *S. brasiliensis*. A detailed study of the genus’ distribution will likely reveal many new taxa. It is essential to highlight that Pennington (1990) pointed out various aspects of *S. brasiliensis* that indicate the importance of further studies: a) the species has two varieties, *S. brasiliensis* var. brasiliensis and *S. brasiliensis* var. *gracilis*, the latter characterized by much smaller leaves, and b) *S. brasiliensis* forms a complex that includes *S. inflexus* and *S. wurdackii*. Pennington (1990) mentioned that it was not possible to differentiate the two named varieties because variation was continuous. Instead, *S. inflexus* and *S. wurdackii* can be distinguished by their pubescent pedicels (i.e. *S. inflexus*) and leaves (i.e. *S. wurdackii*). Moreover, 10 years ago, in the “Manual de plantas de Costa Rica”, two new species of *Sarcaulus* were proposed but not properly described and remained unnamed (Morales 2015). These cases indicate that more detailed studies of field characters and molecular phylogenetics are required to elucidate the diversity in this genus.

Moreover, *S. paujilensis* differs from other taxa in the genus in the characters discussed in the diagnosis and in the following key.

##### Additional specimens examined.

**Colombia** • Boyacá. Municipio Puerto Pinzón, Reserva Proaves El Paujil, Sendero principal hacia barranco, 6°03' 01" N, 74° 15' 51" W, 200 m, 23 oct. 2024, fr., *A. F. Majin-Ladino, L.D. Rodríguez, J. Briceño & M. Mahecha 235* (UMNG-H); Municipio Puerto Pinzón Reserva Proaves El Paujil, Sendero La Guinea hacia El Minero, 6°01'31"N, 74°12'6"W, 380 m, 7 Feb 2025 [fr]. *A. F. Majin-Ladino, Adriana L. Torres, Aura M. Jurado & Jesús Sánchez 288* (UMNG-H. HUA); Municipio Puerto Pinzón, Reserva Proaves El Paujil, Sendero El Caño, 6°02'46"N, 74°16'4"W, 210 m, 12 Feb 2025 [fr]. *A. F. Majin-Ladino, Adriana L. Torres, Aura M. Jurado & Jesús Sánchez 339* (UMNG-H, HUA).

### Key for the species of *Sarcaulus*

Modified from Pennington 1990.

**Table:** 

1a	Leaves pubescent on the abaxial surface	**2**
2a	Young shoots finely appressed puberulous, trichomes greyish-brown; leaves 8.3–14.7 cm long, secondary veins 9–13 pairs, leaf indumentum closely appressed puberulous below (whitish trichomes) abaxially, becoming sparse with age; pedicel 3–4 mm long; corolla ca. 2 mm long	***S. vestitus* (western Amazonian, Brazil, Ecuador, Peru; La Macarena range, Colombia)**
2b	Young shoots densely short brown-pubescent, trichomes grey-brown to whitish-grey; leaves 20–23 cm long, secondary veins 14–15 pairs, leaf indumentum uniformly and closely appressed puberulous (brown trichomes) abaxially; pedicel 1.2–1.6 cm long; corolla ca. 3 mm long	***S. wurdackii* (Peru)**
1b	Leaves glabrous on both surfaces	**3**
3a	Pedicel 1.5–4 mm long; corolla and staminodes glabrous; fruit oblate or ovoid	**4**
4a	Leaves 12–16.6 × 3.7–5.5 cm, elliptic to narrowly oblong-elliptic, apex narrowly attenuate or acuminate, base acute to narrowly attenuate, inter secondaries veins often extending to near the margin; petiole 0.8–1.5 cm long; inflorescences 5-many-flowered; flowers unisexual (plant dioecious); sepals ca. 2.5–3 mm long; stamens fixed at the top of the corolla tube; staminodes 0.5–1 mm long; ovary minutely adpressed puberulent; fruit oblate	***S. oblatus* (Ecuador)**
4b	Leaves 21–26 × 6.0–7.1 cm, oblanceolate to oblong, apex shortly acuminate, base acute, inter secondaries veins absent; petioles 1.7–2.5 cm long; inflorescences-2-flowered; flowers bisexual; sepals 5–6 × 4–5 mm; stamens fixed at ca. 1 mm from the petal base; staminodes ca. 2 mm long; ovary shortly sericeous; fruit ovoid	***S. paujilensis* (Middle Magdalena Valley in Colombia)**
3b	Pedicel 10–36 mm long; corolla usually with some indumentum on both surfaces, staminodes appressed puberulous outside; fruit subglobose to ellipsoid	**5**
5a	Pedicel 1–1.5 cm long; corolla 2.5–4 mm long, tube strongly carnose	***S. brasiliensis* var. brasiliensis (Costa Rica to Bolivia)**
5b	Pedicel 2–3.6 cm long; corolla 4–5.5 mm long, tube weakly carnose	**6**
6a	Leaves elliptic, elliptic-lanceolate or oblong-elliptic; pedicel glabrous; corolla lobes glabrous outside, staminodes triangular or broadly ovate, upright, appressed puberulous outside	***S. brasiliensis* var. *gracilis* (Peru, Chocó Region)**
6b	Leaves broadly elliptic, oblong-elliptic or oblong-lanceolate; pedicel appressed puberulous; corolla lobes often sericeous outside; staminodes broadly triangular, strongly inflexed, appressed pubescent outside	***S. inflexus* (central Brazilian Amazonia)**

## Supplementary Material

XML Treatment for Sarcaulus

XML Treatment for Sarcaulus paujilensis

## Additional information

### Artificial Intelligence (AI) use

The authors accept full responsibility for the content of the manuscript, including the disclosure of any use of AI.

No AI tools were used in the preparation of this manuscript.

### Author ORCIDs

M.A. Jaramillo https://orcid.org/0000-0002-6539-4149

T.D. Pennington https://orcid.org/0000-0003-3610-0946

G.A. Aymard-Corredor https://orcid.org/0000-0001-9405-0508

A.F. Majin-Ladino https://orcid.org/0009-0009-5013-617X

### Data availability

All of the data that support the findings of this study are available in the main text.
